# The Irish bTB eradication programme: combining stakeholder engagement and research-driven policy to tackle bovine tuberculosis

**DOI:** 10.1186/s13620-023-00255-8

**Published:** 2023-11-23

**Authors:** Eoin Ryan, Philip Breslin, James O’Keeffe, Andrew W. Byrne, Karina Wrigley, Damien Barrett

**Affiliations:** Department of Agriculture, Food and the Marine, Kildare St, Dublin, 2 Ireland

**Keywords:** Bovine, Tuberculosis, Ireland, Badger, Culling, Vaccination, BCG, Tuberculin, Testing

## Abstract

A new Irish bovine tuberculosis (bTB) eradication strategy was launched in 2021. The strategy was formulated following extensive discussions with stakeholders, formal reviews of several aspects of the existing bTB policy and relevant inputs from the latest scientific research projects. A stakeholder discussion body, the TB Forum, had been established in 2018 and this continues under the new strategy, supported by three working groups (scientific, financial and implementation). The strategy sets out actions to address cattle-to-cattle and badger-to-cattle bTB transmission, along with actions to improve farm biosecurity and empower farmers to make their own choices to reduce bTB risk.

Large scale vaccination of badgers has been rolled out under the new strategy, with over 20,000 km^2^ covered by the vaccination programme and 6,586 badgers captured in vaccination areas in 2021. Vaccination efforts have been complemented by intensive communications campaigns, including a web enabled software application (“app”) enabling farmers to report the location of badger setts.

Cattle which test inconclusive to the tuberculin skin test have been re-tested using a gamma interferon blood test since April 2021, enabling truly infected cattle to be identified more effectively due to the higher sensitivity of this test. An enhanced oversight process has been put in place for herds experiencing extended or repeat bTB breakdowns. Whole genome sequencing is being used to investigate links between breakdowns, with the results supporting operational decision making in case management.

Communications, including biosecurity advice, are co-designed with stakeholders, in order to improve their effectiveness. A programme involving veterinary practitioners providing tailored biosecurity bTB advice to their clients was established in 2021 and was rolled out nationally during 2022.

A core element of the new strategy is the continual improvement of policies in response to changing bTB risks, informed by scientific research and then implemented with stakeholder consultation.

## Introduction

Bovine tuberculosis (bTB), caused by *Mycobacterium bovis*, continues to pose a significant animal health challenge in Ireland. The objective of this paper is to describe advances within the Irish bTB eradication programme in recent years and to set out the structural relationships between scientific research, stakeholder attitudes and policy development. The paper also seeks to provide a wider historical perspective on the Irish bTB programme, putting the recent changes and strategic developments in context.

Current bTB levels in Ireland impose significant direct and indirect costs on the public and private sectors and are a threat to Irish export market access. In 2021, national herd incidence was 4.33%, with 20,931 cattle identified as TB test reactors. Direct costs in 2021 were estimated at €105 m, with €67 m paid by the State, an estimated €35 m paid by farmers and €3 m by the EU; these figures do not include indirect costs or production losses. The costs of bTB in Ireland are not confined to financial issues; restrictions applied to infected herds can cause significant emotional distress to herdowners [1].

An eradication programme for bTB is required under EU legislation, with the rules for countries or zones where disease is present set out in Regulation (EU) 2016/429 (the Animal Health Law) and Commission Delegated Regulation 689/2020 (which sets out the specific requirements for bTB). The Irish bTB programme applies these rules, as well as additional national requirements relevant to the epidemiological context in Ireland.

The Irish bTB eradication commenced in 1954, with tuberculin skin testing used in all herds to identify infected cattle, which were then removed and slaughtered. At that point, it was estimated that 80% of herds and 17% of cattle (22% of cows) were infected [2]. By 1965, Ireland had achieved a status that would allow its cattle to be traded in the EEC when its subsequent application for entry was eventually successful in January 1973, consistent with the requirements of EEC Directive 64/432 [2]. Throughout the 1970 and 1980 s, however, progress stalled. In 1988, an executive agency was established to drive the bTB eradication programme, named the Eradication of Animal Diseases Board, or ERAD. A stronger focus was introduced on the use of scientific research to inform policy, along with the development and use of improved metrics and stricter controls [3]. By 1992, when ERAD was integrated into the Department of Agriculture veterinary service, bTB levels were higher but this was considered to be a reflection of more successful efforts to detect disease, rather than an increase in bTB per se. Recommendations were made regarding the future strategic direction of the Irish bTB programme, emphasising the need to address the role of badgers in spreading bTB; the requirement for a blood test to complement or replace the skin test; stricter controls to impede cattle-to-cattle transmission; the importance of enabling farmers purchasing cattle to know the TB history of the source herds to reduce the unwitting introduction of new bTB infections; an emphasis on improved biosecurity at farm level through education and awareness raising; and an overarching need for ongoing scientific research to inform future policies [4]. These recommendations informed much of the strategic direction of the Irish bTB programme in subsequent decades [5–7].

A major bTB research programme was established in 1991, principally involving researchers employed within the Department of Agriculture, Food and the Marine and University College Dublin School of Veterinary Medicine, with the aim of providing an evidence base for new policies and strategic decisions [8]. Research was carried out into the role of badgers in bTB epidemiology in Ireland and into the effectiveness of interventions such as badger culling [9–11]. A research programme accompanied the introduction of a badger culling policy, which included work to investigate the impact of culling on several aspects of badger eco-epidemiology including: TB levels in badgers [12], badger population abundance [13, 14], movement [15], and wildlife management [16, 17]. The research programme also supported work not directly related to culling policy aimed at informing a wider understanding of badger ecology in Ireland [18–25].

A significant and ongoing research programme on the use of Bacille Calmette-Guerin (BCG) vaccination in badgers to reduce the risk of badger-mediated bTB transmission to cattle was a key element of this overall research programme [7]; this research is discussed in more detail below.

The challenge of improving diagnostics for bTB was also a focus of much research. This included work on the tuberculin test [26–28], the interferon-gamma blood test [29, 30] and the potential role of ELISA tests [31, 32]. Research on the role of cattle movements and surveillance was also carried out and will be discussed later.

By 2016, bTB levels in Ireland had reached an all-time low, with herd incidence at 3.27%. However, the removal of milk quotas in 2015 prompted the expansion of the dairy sector in Ireland. Dairy herds, particularly the larger ones, have many risk factors for bTB, and thus have a higher disease incidence compared to other cattle enterprises. The expansion of the dairy sector was therefore associated with an increase in bTB. Consequentially, bTB levels rose each year until they peaked at 4.38% in 2020. The confluence of improved scientific understanding, the availability of new tools, increasing disease levels, and concerns about the ongoing costs and burden of bTB prompted an initiative to renew the Irish bTB strategy in 2018.

## The framework for the development of a renewed Irish bTB eradiction strategy

The TB Forum was established in 2018 to discuss bTB and make recommendations to the Minister for Agriculture, Food and the Marine on additional measures which could be implemented to reduce bTB levels and further the drive towards eradication. The Forum was composed of a range of stakeholders and submitted a report on its recommendations in 2019. This report, the policy analysis document which informed its development, and the minutes of the TB Forum meetings, can be accessed at www.bovinetb.ie [33]. Two reports were commissioned by DAFM at the request of the TB Forum; one on the costs and benefits of the bTB eradication programme, and the other on the compensation arrangements for owners of reactor cattle [33]. Following further discussions with the TB Forum, DAFM published a new TB strategy in 2021 setting out a range of additional actions to reduce bTB transmission and drive levels towards eradication [34].

The framework for considering the range of bTB issues was for policies to be reviewed and discussed at the TB Forum and also subjected to ongoing scientific research and policy analysis. This was often an iterative process, with the results of research informing an updated policy analysis, leading to proposals which, if implemented, would then be the subject of further scientific research. These steps were not necessarily sequential but could progress in parallel, overlapping temporally. The TB Forum provided a vehicle for the frequent exchange of policy information, scientific results, and stakeholder opinions. The policies which were introduced or updated as a result of this process, and the manner of their implementation, were then the subject of ongoing discussion at the TB Forum. In this way, a network approach to policy development was employed, enabling stakeholders to participate in the co-design of policies and the consideration of challenging choices between policy alternatives.

## Badger vaccination

The idea of using Bacille Calmette Guerin (BCG) to vaccinate badgers and thus reduce badger-to-cattle transmission of *M bovis* has been the subject of a considerable body of scientific research in recent decades, particularly in the UK and Ireland. Many studies investigated the potential of orally-administered BCG to generate protective immunity in badgers [35–38]. Despite initially promising results, an oral vaccine for badgers was not progressed to production stage and this line of research ceased in Ireland.

A major field trial was carried out in Co. Kilkenny on field vaccination of badgers [39, 40]. A separate major study, termed the non-inferiority badger vaccine trial, evaluated injectable BCG vaccination of badgers in relation to cattle bTB levels in seven study areas across Ireland [41].

Research on vaccination was complemented by the badger ecological research mentioned above, enabling the design of a vaccination programme which could be implemented nationally in the field. The knowledge base was further progressed by studies carried out in Northern Ireland which examined similar topics in a similar ecological situation [42, 43].

In 2018, routine vaccination of badgers using injectable BCG was established as policy within the Irish bTB eradication programme. Initially, vaccination was carried out in areas which had been part of the field research programmes, with incremental expansion to additional areas over time. The 2019 Programme for Government included a commitment to extend the badger vaccination programme nationwide and end badger culling as soon as possible, consistent with the best scientific and veterinary advice.

**Fig. 1:**
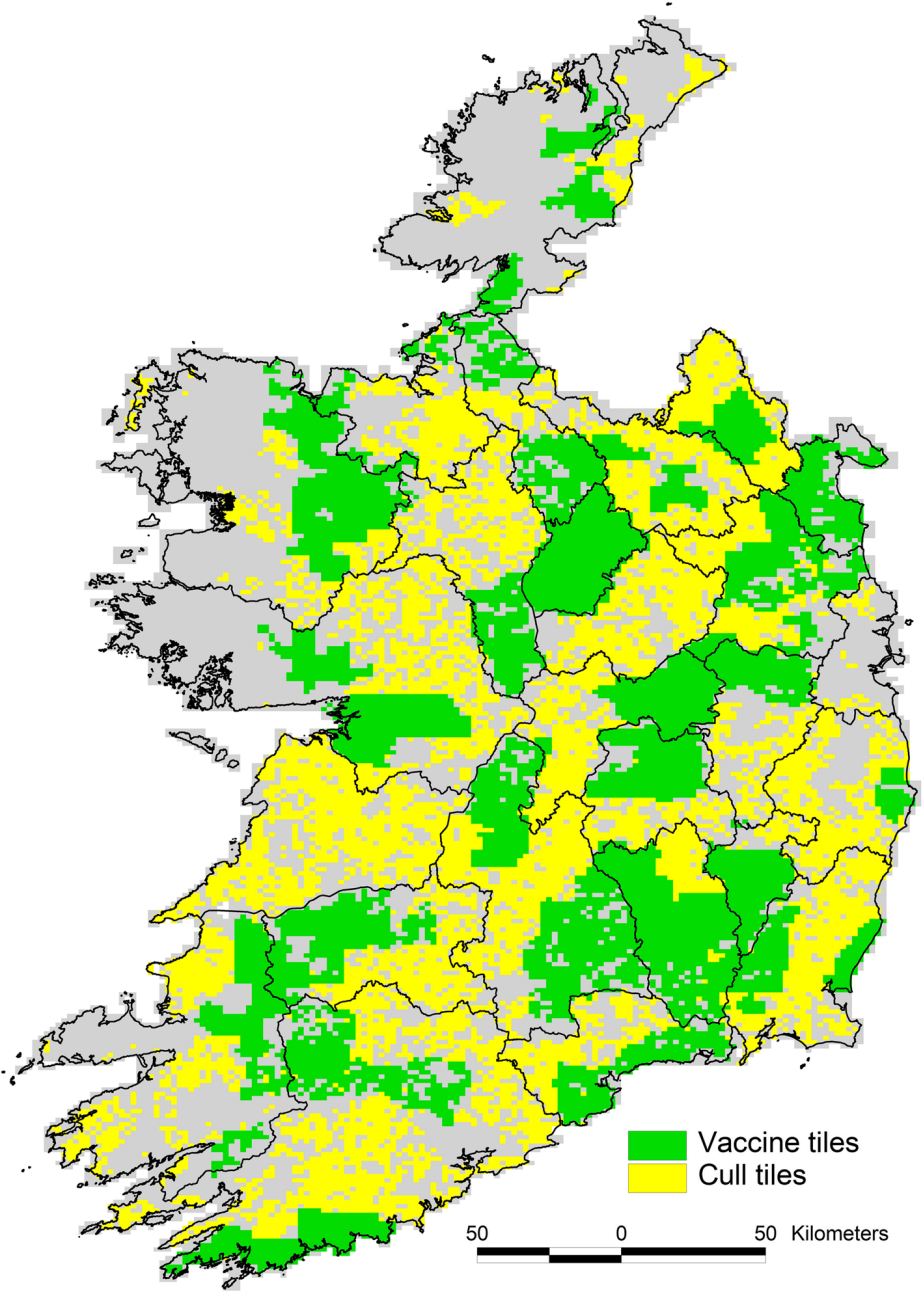
Areas where badger vaccination was carried out in Ireland in 2021. Yellow areas are where culling is carried out; green areas are where vaccination is carried out. A tile is an area of 2 km by 3 km which is the operational management unit for badger culling and vaccination in Ireland

By 2021, over 20,000 km^2^ in Ireland was designated a badger vaccination area; this is more than half of the total area on which the DAFM wildlife unit operates. In these areas, vaccination is the default, although culling may still be carried out where required for epidemiological reasons. In 2021, the DAFM wildlife unit captured 6,586 badgers in vaccination areas, of which 3,958 were then vaccinated (badgers captured which are found to have been previously vaccinated are not re-vaccinated), and captured 5,868 in culling areas (Fig. 1).

Two innovations were introduced to support this policy. In areas where concerns arose that infection prevalence in badgers may be high, penside tests (made by Chembio (3661 Horseblock Rd, Medford, NY 11,763, USA) and Ingenasa (Calle de los Hermanos García Noblejas 39, 28,037 Madrid Spain)),) were used on badgers prior to vaccination; badgers which tested positive were culled and sent for post-mortem examination and culture. This work is ongoing and the results are expected to be published when complete. A second innovation was the development of a mobile phone app whereby farmers could report the location of badger setts to DAFM, to improve the population penetration of the vaccination programme, a key metric for effective badger vaccination [44]. This app was launched in October 2021 and resulted in over 500 sett locations being reported to DAFM in the first year of operation; the app is available to download at www.bovinetb.ie.

The effectiveness of the badger vaccination programme and its impact on local transmission dynamics are the subject of an ongoing research project (Barber A and Chang Y, personal communication). The interim results of this work are regularly presented to stakeholders in the context of the TB Forum, providing for a degree of stakeholder confidence in the programme and demonstrating the continued commitment to evaluating and reviewing policies, including the badger vaccination programme.

## Animals which test inconclusive to the SICCT test

Cattle which test inconclusive to the SICCT test were required to be retested 42 days later, or slaughtered and subjected to laboratory examination or a balance of herd test in line with the requirements of Directive 64/432. If the repeat test disclosed them as inconclusive again or as positive, they were deemed reactors; while if negative, they were deemed to have passed the retest. Research had confirmed that in Ireland, such cattle which pass the retest continue to pose an elevated risk of being infected [45, 46]. Based on this research, policy was amended in 2011 such that inconclusive cattle were restricted to the herd of disclosure for life, with advice given to the herdowner that these cattle posed a risk and ought to be culled.

At the TB Forum, stakeholders raised concerns about the ongoing risk posed by such retained inconclusives; while confined to the herds of disclosure for life, they could still initiate a repeat breakdown within those herds which may spread onwards. In response to these concerns, an updated policy on inconclusives was developed and implemented, informed by the earlier research. Policy analysis revealed that approximately 3,500 cattle annually tested inconclusive, with roughly 20% of these failing the retest.

The new policy required cattle which tested inconclusive to the SICCT test to be subjected to an interferon gamma blood test within three weeks. If positive, the animal was deemed a reactor and removed. If negative, the animal was still required to undergo a further SICCT test. The interval for this repeat skin retest was increased from 42 to 60 days in order to counter the desensitising effect of tuberculin injection. If the animal passed this, it would require a further interferon gamma test six months later.

Other related policy changes included the mandatory removal of any previously disclosed historic inconclusives (i.e. an animal which had, in the past, tested inconclusive and then re-tested negative and thus was allowed to remain in the herd) from a herd if one or more reactors was disclosed at a test; any cattle newly testing inconclusive to be deemed reactors if one or more standard reactors were disclosed at the herd test; and where four or more cattle tested inconclusive at a herd test with no standard reactors, all four were to be deemed reactors and removed and the herd restricted. In 2021, a letter was sent to the owners of all historic inconclusives, advising them to cull these cattle if they wished to reduce the bTB risk to their herd.

In the first full year of operation of the new policy, 61% of inconclusive cattle which were interferon gamma tested were positive and thus removed as reactors, with the herd restricted. Within six months of the issuing of the advice letter on historic inconclusives by DAFM referred to above, 26% of historic inconclusive cattle had been slaughtered (4,232 of these historic inconclusives were alive in September 2021 when the letters were issued versus 3,133 alive in April 2022). The number of cattle reported as tested inconclusive to the skin test was 1,497 in the first full year since the new policy was introduced, a major reduction compared to the roughly 3,500 reported annually in years prior to the new policy.

## Extended and recurrent breakdowns

The subject of herds which experience extended and/or repeated bTB breakdowns has been the focus of considerable epidemiological research in Ireland [47–51]. The bTB programme has distinguished between breakdowns on the basis of the number of reactors disclosed, with more severe restrictions and disease investigation measures applied to breakdowns with three or more standard reactors. Table 1 shows the number of standard SICTT reactors per breakdown in Ireland from 2014 to 2021.

**Table 1:** The number of standard SICCT test reactors per bTB herd breakdown in Ireland from 2014–2021. Breakdowns with zero standard reactors are those identified by at least one animal with a positive factory lesion or those with an inconclusive animal which tested repeat inconclusive or which had a non-standard reactor detected in the context of a risk-based herd test, and which had no standard reactors during the course of the subsequent breakdown

	**0**	**1**	**2**	**3**	**4 to 9**	**10 to 15**	**> 15**
**2021**	1,557	1,444	657	325	558	98	70
**2020**	1,333	1,594	638	349	678	123	85
**2019**	1,106	1,390	598	300	497	117	87
**2018**	1,060	1,383	553	264	470	89	78
**2017**	1,106	1,391	521	244	435	98	101
**2016**	1,022	1,335	497	212	440	93	96
**2015**	1,080	1,411	530	239	398	99	77
**2014**	1,148	1,487	556	271	452	103	102

Stakeholders are aware of the differences in seriousness between breakdowns based on number of reactors and length of restriction, and the subject of how better to manage such cases was discussed at the TB Forum. Informed by the research findings cited above, case management policy was updated to emphasise the progressive removal of all sources of bTB risk from extended and/or repeat breakdown herds. For example, if such a herd is not going clear, cattle whose test result is deemed “severe inconclusive” are removed, followed by cattle with a bovine bias in the SICCT, followed by cattle considered at higher risk due to their history of exposure. The policy discussions prompted a specific research project on the relationship between the bovine tuberculin response and future risk [52], the results of which enabled an iterative updating of the evidence base for the policy and informed further discussions at the TB Forum.

This topic is closely linked to the broader issue of effective breakdown management, where again new research has informed policy considerations. For example, research on faecal shedding of *M. bovis* in reactor cattle [53] underpinned a policy review on the management of slurry from infected herds, providing new data with practical utility and relevance on a case management issue which had previously been the subject of a very limited number of studies.

### Surveillance for bTB in cattle at slaughter

All cattle slaughtered in Ireland are subjected to ante- and post-mortem inspections by a veterinarian, as required under EU food hygiene legislation. This includes checks for lesions which are suspected of being caused by bTB. Such lesions, termed “suspect factory lesions” in Ireland, are sent for laboratory testing and, if positive, the infected herd is restricted. Approximately one third of breakdowns in Ireland annually are first identified through surveillance at slaughter. Given its critical role in bTB surveillance in Ireland, this area has been the subject of a number of research studies [54, 55], with a recently published paper evaluating data from 2014 to 2018 [56]. The results of these studies have informed policy development and the ongoing management work of monitoring the delivery of this aspect of the bTB programme and delivering training to those carrying out the inspections.

### Diagnostic tests for detecting bTB infection

The primary diagnostic test for detecting bTB in Ireland is the SICCT. The characteristics of this test make it the most suited option for a mass-screening programme, given its very high specificity [32]. However, its relatively low sensitivity has been recognised as being a challenge for an eradication campaign [4, 7], thus driving interest in other diagnostic tests which could also play a role in the programme. At the request of the TB Forum, this topic was recently reviewed by the Scientific Working Group of the TB Forum, whose opinion was presented to stakeholders to inform discussions on how best to make use of existing tests; this opinion has been published online [57].

The use of the interferon gamma test was introduced as policy for serious bTB breakdowns (defined as four or more reactors) in 2015, following several years of research in Irish field conditions [29, 32]. The cut-off for positives was set at [bovine optical density] – [avian optical density] > 0 = positive, with samples exceeding this threshold deemed positive and the cattle compulsorily removed as reactors [30]. This cut-off was lower than that described in the manufacturer’s instructions, with the aim of increasing sensitivity. The data was reviewed and analysed after three years [30], and the cut-off was subsequently revised to [bovine optical density]-[avian optical density] > 80 = positive; this was still lower than the manufacturer’s instructions (B-A > 100 = positive) and balanced sensitivity and specificity in the context of Irish field conditions. Three years later, the data were again analysed, examining the subsequent fate of cattle which would have been deemed positive under the old criteria, but which had been deemed negative under the new conditions (this analysis will be published separately). These results were used to inform another review of the cut-off; based on the data, the decision was made to retain the cut-off at B-A > 80 and not to change further at that time. This process illustrates the role of regular, planned evaluation studies carried out on several years of data in ensuring that policy reviews are supported by robust scientific research, in order to make the optimum decisions.

The tuberculin used in the programme has long been recognised as a key element requiring quality control and ongoing evaluation, with research showing the importance of using tuberculin of sufficient potency in the context of an overall quality control programme on bTB testing [26–28].

## Communications

Effective communication is crucial to the success of disease eradication campaigns, and the stakeholders at the TB Forum had signalled that improving communications to herdowners should be considered a priority. This was supported by research carried out on the attitudes of Irish farmers to the bTB programme [1].

**Fig. 2:**
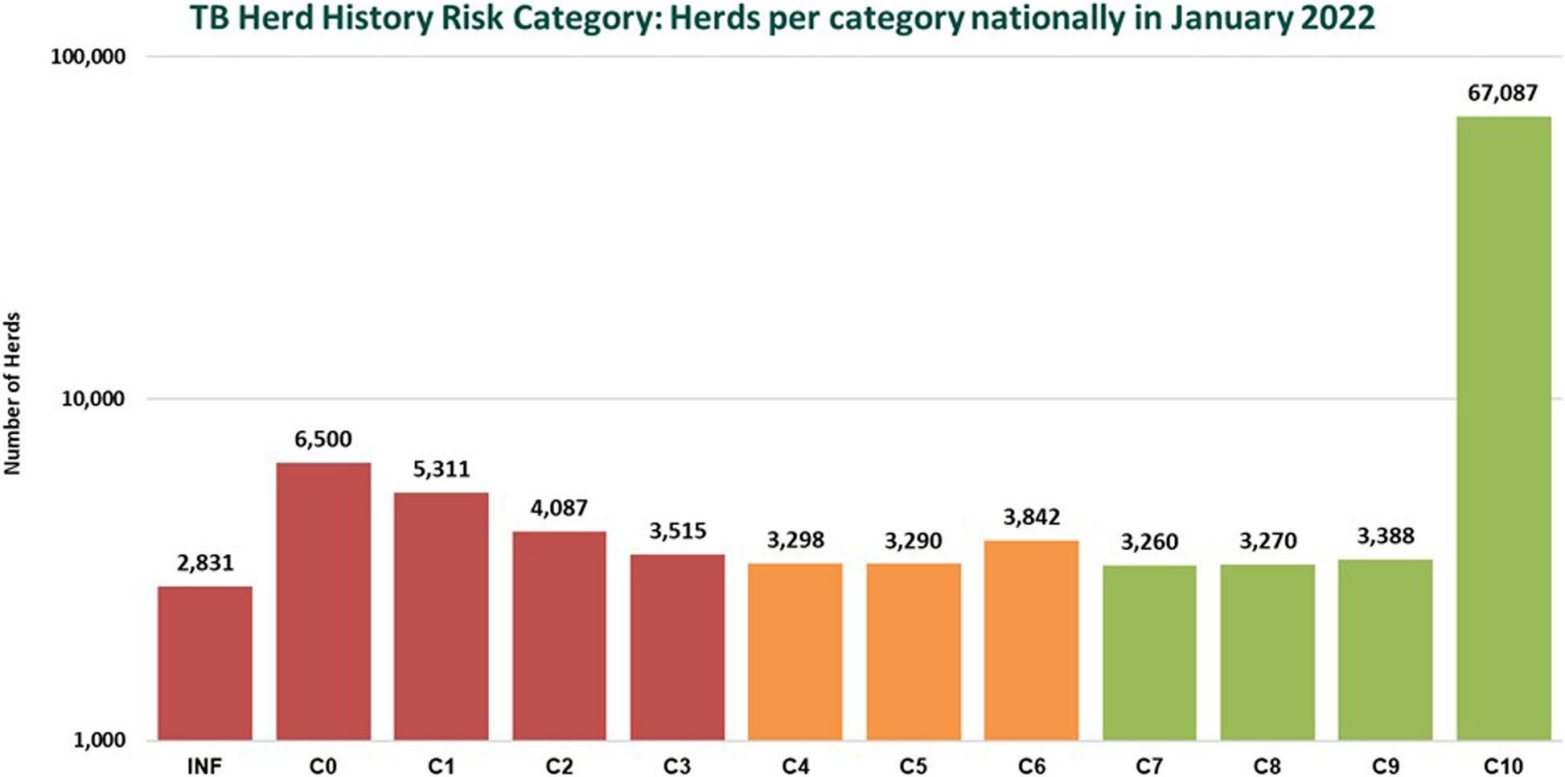
Bar chart showing the number of cattle herds in each TB herd history risk status category in January 2022. Inf = infected. Herds clear of bTB for 10 years or more are all classified as C10

The subject of risk communication can be particularly challenging; in 2020, each cattle herd was assigned a TB herd history risk (TBHHR) status, which described if a herd was clear (C) or infected (INF), how many full years it had been clear and the number of bTB breakdowns in the preceding 10 years. For example, a TBHHR score of C4(2) means a herd has been clear for four full years and has had two bTB breakdowns in the past ten years. Figure 2 shows the number of herds in each category in January 2022. Stakeholder organisations at the TB Forum expressed dissatisfaction with herdowners being informed of their own TBHHR status, indicating that further work on risk communication was needed.

The conflict between the interests of the buyer in avoiding introducing potentially bTB-infected cattle into their herd versus the interests of the seller of bTB-exposed cattle in maximising the price paid has been addressed by previous reviews of bTB in Ireland, including as far back as 1991 [3] and a 1994 report by the Public Accounts Committee [58], which stated that “until the interests of the buyer are prioritised over those of the seller”, bTB eradication would remain a challenge in Ireland. The continuing diversity of opinions among stakeholders on this point illustrates the difficulties of risk communication in the context of bTB, but also highlights the opportunities which may be presented by continuing engagement with stakeholders. To develop these opportunities, recognising the range of viewpoints, a communications sub-group of the TB Forum was established, to ensure that future communications to herdowners on bTB issues are made more effective through using a co-design process with farming organisations.

The introduction of a biosecurity advisory module on bTB which can be delivered by veterinary practitioners to their clients, funded by DAFM through Animal Health Ireland as part of their Targeted Advisory Service on Animal Health [59], has enabled veterinary practitioners to strengthen their role in the bTB programme. Veterinary practitioners carry out the vast majority of SICCT testing in Ireland and are also a vital, and trusted, source of advice and information for farmers, with a deep knowledge of their clients’ herds and an understanding of the particular dynamics at play in each case.

These actions recognise the complex challenge of engaging with stakeholders on risk communication and advocating risk-reducing behaviour, which has been identified as an important area yet one in which much work remains to be done [60–63].

Other communications efforts established in recent years to engage with farmers include the participation of DAFM bTB team members in many public meetings across Ireland, media engagement including podcasts, radio and television, creating a series of youtube videos and leaflets on specific topics of recurring interest (available to view on www.bovinetb.ie) and partnering with other agricultural and veterinary advisory bodies to deliver training and information.

### Deer and activities disruptive to wildlife

The role of deer in the epidemiology of bTB in Irish cattle is a subject which has attracted increasing stakeholder interest in recent years [7]. In response to these concerns, a number of studies were funded by DAFM, in addition to laboratory testing of deer submitted to Regional Veterinary Laboratories for bTB. It was found that isolates of *M. bovis* from cattle, deer and badgers in Co. Wicklow were very closely related, indicating transmission within and between species [64]. A modelling study based on reported hunting returns and cattle bTB data indicated a potential association between Sika deer numbers and cattle bTB levels in Co. Wicklow [65].

In response to stakeholder concerns, the policy on deer and bTB was updated such that, if herdowners in an area were concerned that deer locally may be infected with bTB and playing a role in spreading disease to cattle, they could submit deer carcases to DAFM Regional Veterinary Laboratories, which would then test those carcases for bTB free of charge and report the results locally and to the TB Forum. This work was not, therefore, a random sample of deer; rather, only deer in areas where there were thought to be significant bTB levels in cattle were submitted for testing. Between 2016 and 2020, 35 of 272 (12.8%) of deer submitted from Co. Wicklow were positive for *M bovis*, while 10/467 (2.1%) of deer submitted from the other 25 counties of Ireland were positive.

Stakeholders at the TB Forum also conveyed the concerns of some of their members that activities which could disrupt wildlife, specifically the construction of roads and the clear-felling of forestry, could lead to an increase in local bTB levels in cattle. Epidemiological research was thus carried out into these questions, with the results shared with stakeholders. Researchers reported evidence of an increased bTB risk in cattle being consequent to, not just coincident with, road construction, and hypothesised that perturbation of badger populations may have provided the mechanism for this effect [66]. Another study found evidence of badger territoriality being maintained during a large road construction project, where the new road was along a similar route to the older road it was replacing [25]. Taking account of these findings, policy was amended to enable badger vaccination in advance of major road or infrastructure projects, with a view towards reducing the risk of transmission of *M bovis* from badgers to cattle in the area consequent to the initiation of construction.

In relation to clear-felling of forestry within a wider ecological landscape context and bTB risk, evidence was found of an increased bTB risk following clear felling of forestry [67], but there were significant interactions with local landscape types, specifically the level of natural grassland and mixed forestry, and with time and distance from the clear felling event [68]. Research on the interaction between landscape, ecology and epidemiology of bTB in Ireland is ongoing, building on these results.

The Scientific Working Group of the TB Forum considered the issue of the sources of bTB infection for cattle, including the possible role of deer; this opinion was presented to stakeholders at the TB Forum and has been published online [69].

### Molecular epidemiology and the use of whole genome sequencing

The use of whole genome sequencing (WGS) has become widespread in bTB epidemiology in recent years [64, 70, 71], providing insights into broader patterns of epidemiology and also into specific outbreaks. Since 2021, increasing numbers of *M bovis* cultures are being analysed using WGS in Ireland, principally isolates from cattle and badgers but also from other species such as deer, pigs and alpacas. The results are used to generate a wider national perspective on the distribution of *M bovis* strains in Ireland, and also to inform specific operational case management decision making and to address particular epidemiological queries at local level. The use of WGS as a tool to support epidemiological investigations and case managements illustrates how technical advances, when linked to the field management of cases, can provide more evidence for the case manager to take into account, with the objective of improving decision making. A detailed analysis of the results of this initiative will be reported separately. The scientific working group of the TB Forum has considered how WGS can best be used to support improved effectiveness in the Irish bTB programme, and their guidance will inform policy development in this area.

### Adapting the Irish bTB eradication programme during the Covid19 pandemic

The introduction in March 2020 of societal restrictions to protect public health in relation to Covid19 in Ireland necessitated a rapid consideration of how the Irish bTB eradication programme could be adapted such that the public health rules were complied with while protecting animal health and enabling the continuation of cattle trade, considered a vital part of the Irish food supply system.

Throughout the pandemic period, there was ongoing and frequent communication between the DAFM bTB team and stakeholders, particularly those representing farmers, veterinarians, and cattle marts. As the public health restrictions changed over the course of the pandemic, so too did the changes applied to the bTB programme, following discussions on each occasion with stakeholders. This illustrates the value of setting in place structures, such as the TB Forum, for ongoing engagement between policy makers and stakeholders.

At the start of the pandemic, DAFM’s high containment TB laboratory became a site for Covid19 testing. Although the consequent interruption to bTB testing turned out to be brief, concerns arose at the time that there could be a prolonged interruption to bTB laboratory culture capacity, due to the use of the laboratory for Covid19 testing and due to the risk of the highly experienced laboratory staff becoming unavailable if they became infected. The question arose of how to manage herds which had cattle with suspect factory lesions, if those lesions could not be cultured and tested for *M bovis*. A study was rapidly carried out on the risk factors related to the likelihood of a herd with a suspect lesion in a slaughtered animal subsequently having a bTB breakdown [72]. Based on this research, a new policy was drawn up and discussed with stakeholders, enabling the effective management of this risk through having regard to the wider epidemiological factors.

Guidance was provided to herdowners and veterinarians in relation to conducting the SICCT while adhering to the public health rules. Herd tests could be postponed for up to 28 days past the due date without penalty, to allow for circumstances where one party may have suspected or confirmed Covid19 infection, or where it proved challenging to obtain help from normal sources to assist with the test on the scheduled date. Small calves are usually held by one person while the veterinarian conducted the SICCT (rather than put in a crush, as older cattle are). To allow for social distancing guidelines to be adhered to, a temporary easement was allowed whereby calves aged 42–120 days could be excluded from a herd test if either the herdowner or the veterinarian were of the opinion that they could not be tested while adhering to the Covid19 rules. This easement was removed as Covid19 restrictions and social distancing guidance were changed. A review of the epidemiological considerations and risks related to bTB in calves was carried out to support policy development during this period; this work has been published and describes the topic in more detail [73].

### Future directions of policy development and research

Machine learning approaches to predicting bTB risk have been described [74]. A similar model, using machine learning and the data available in the Irish Animal Health Computer System and Animal Identification and Movement system, has been developed to predict animal-level bTB risk over a future period which can be varied between 180 and 380 days, and it is hoped that this will act as a decision support tool for case managers; this work will be described in detail separately. A number of other research projects are underway in the field of disease modelling and decision support tools for bTB in Ireland. Such models, it is hoped, may support discussions at the TB Forum by enabling policy makers and stakeholders to consider the likely impact of different options for reducing bTB transmission, while also providing an improved understanding of bTB epidemiology in Ireland.

The scientific working group of the TB Forum has completed a consultation on future bTB research priorities with stakeholders from farming, veterinary and research backgrounds. Following the consultation, replies were systematically evaluated and priorities for future research were identified and presented to the TB Forum. These will inform the likely direction of bTB research in Ireland over the coming years.

The wider international context will also continue to inform the development of Irish bTB policy. The bTB programme in Ireland sits within the legal framework of the EU regulations on the eradication of bTB, while the attitudes of trade partners constitute a major consideration for Irish policy makers. Technical and scientific developments in relation to bTB will also influence the Irish programme, with relevant innovations considered regarding the Irish epidemiological context.

## Conclusion

The Irish bTB eradication strategy will continue to change, adapting in response to new risks and new circumstances and seeking to make use of new tools as they become available. The paradigm of developing policy based on scientific research and through engagement and discussion with stakeholders provides a responsive and flexible model for ensuring that the programme can effectively tackle bTB in Ireland, driving towards an ultimate goal of eradication.

## Data Availability

Not relevant.

## Abbreviations

BCG: Bacille Calmette-Guerin
bTB: bovine tuberculosis
DAFM: Department of Agriculture, Food and the Marine
EEC: European Economic Community
EU: European Union
SICCT: Single Intradermal Comparative Tuberculin Test
TBHHR: Tuberculosis herd history risk
WGS: Whole genome sequencing
